# Cat Ownership and Rural Residence Are Associated with Lyme Disease Prevalence in the Northeastern United States

**DOI:** 10.3390/ijerph19095618

**Published:** 2022-05-05

**Authors:** Amanda Roome, Katherine Wander, Ralph M. Garruto

**Affiliations:** 1 Bassett Research Institute, Mary Imogene Bassett Hospital, Cooperstown, NY 13326, USA; 2 Department of Anthropology, Binghamton University (State University of New York), Binghamton, NY 13902, USA; katherinewander@binghamton.edu (K.W.); rgarruto@binghamton.edu (R.M.G.)

**Keywords:** Lyme disease, tick-borne disease, risk factors, risk behaviors

## Abstract

Lyme disease (LD) is the most common vector-borne disease in the USA. Beyond its tick-borne nature, however, risk factors for LD are poorly understood. We used an online questionnaire to compare LD patients and non-LD counterparts and elucidate factors associated with LD. We investigated demographic, lifestyle, and household characteristics and use of prevention measures. Associations with LD were modeled using logistic regression, and average marginal effects were estimated. In total, 185 active or past LD patients and 139 non-patients participated. The majority of respondents were white (95%) and female (65%). Controlling for age, sex, and type of residential area, pet ownership was associated with an 11.1% (*p* = 0.038) increase in the probability of LD. This effect was limited to cat owners (OR: 2.143, *p* = 0.007; dog owners, OR: 1.398, *p* = 0.221). Living in rural areas was associated with a 36% (*p* = 0.001) increase in the probability of LD compared to living in an urban area. Participants who reported knowing someone with Lyme Disease were more likely to wear insect repellant and perform tick checks. This study suggests opportunities for improved LD prevention, including advising cat owners of their increased risk. Although patterns in adoption of LD prevention methods remain poorly understood, concern about LD risk does motivate their use.

## 1. Introduction

Lyme disease (LD), caused by the spirochete *Borrelia burgdorferi,* is transmitted to humans through the bite of an infected *Ixodes scapularis* tick, more commonly known as the deer tick in the Northeastern United States [1,2]. Infection can cause flu-like symptoms, unusual fatigue, joint and musculoskeletal pain, headaches, trouble sleeping, and depression, among other symptoms [3,4,5,6]. If left untreated or not promptly treated, symptoms can progress into serious cardiac and neurological complications that are often debilitating to patients [4,7].

*I. scapularis* is widely distributed in the eastern United States (US); however, the vast majority of Lyme cases in humans occur in the Northeast and Upper Midwest, with an estimated 476,000 cases occurring annually [8]. Densely populated suburban and peri-urban regions throughout the Northeast are situated in areas with high LD transmission [9], where once contiguous forests are broken up by roads and manmade structures into smaller microecologies, promoting the propagation of tick populations and increasing the prevalence of *B. burgdorferi* in reservoir species [10,11,12].

Environmental and ecological factors influencing LD transmission have been extensively studied [2,9,10,13,14,15]. These studies have found that forest fragmentation [typically through suburban development] decreases species biodiversity, which increases white-footed mouse populations, thereby increasing the density of infected nymphal ticks in human environments [2,10,13,14,15]. Rural living has been found to increase the risk of contracting LD, as does the activity of clearing brush from residential properties that border wooded areas [9]. Other identified risk factors for LD include outdoor activities such as hiking and woodcutting [16] and the number of hours individuals spend outside [17]. Additionally, household pets, namely dogs, can act as a sentinel species for LD and leave their owners at risk by transporting ticks into residences [18].

Although health agencies in many states and counties provide public guidance for preventing tick bites, strategies for prevention could be better targeted with more complete information about the efficacy and acceptability of these methods and public perceptions of tick bite risk. Research investigating the efficacy of prevention methods suggests that protective clothing may actually increase LD risk; as do behaviors such as sitting on logs or grass and contacting leaf litter [17,18,19]. Risk perceptions seem to impact precautionary behaviors in a somewhat counterintuitive manner: the more confident a person is that they will find a tick on themselves, the less likely they are to take precautionary measures [20,21].

Much of the existing research on LD has been conducted on the West Coast, where the primary vector for *B. burgdorferi* is a different species of tick than in the Eastern US, *Ixodes pacificus.* [16,22]. An up-to-date assessment of risk factors for LD in the Northeastern US, where LD prevalence is high, can aid health agencies in developing and improving intervention and prevention strategies. The purpose of this study was to identify demographic and behavioral risk factors for Lyme disease in the Northeast and describe factors associated with the use of recommended prevention and protection measures (e.g., insect repellant and checking oneself for ticks).

## 2. Materials and Methods

A 66-question survey was developed to assess risk factors for LD and lifestyle changes as a result of LD (Supplementary Materials: Survey). Recruitment was targeted to both LD patients and non-LD patients. The survey was advertised via social media (Facebook), emails to LD support groups in New York, New Jersey, and Pennsylvania, and LD conferences, educational workshops, and public presentations. Interested participants were directed to an online link to the survey or to contact the first author working under an approved IRB protocol from Binghamton University (SUNY) in Binghamton, NY, USA for a paper copy of the survey or a link to the online survey (via Google Forms).

Consenting participants were directed to a two-part survey. The first portion inquired about individual and household characteristics and physical activity, social, eating, and sleep patterns. Participants were asked about their mood, healthcare utilization, LD history, and tick bite prevention practices. Participants were also asked if they knew someone with LD and the nature of their relationship (e.g., immediate familial relation or non-immediate relation). Participants who reported having been diagnosed with LD by a physician continued on to the second section of the survey, which gathered information on participants’ LD experience: date of diagnosis, treatment, symptoms and symptom duration, frequency of physical and social activity before and after diagnosis, and changes in eating, sleep, mood, doctor visits, and overall health patterns. Questions were also asked regarding prevention practices before and after an LD diagnosis.

Logistic regression models were estimated to assess risk factors for LD and factors associated with use of prevention measures using STATA Version 15 software, StataCorp LLC (College Station, TX, USA). Both odds ratios (OR) and average marginal effects (AME) were estimated from logistic regressions: AME can be interpreted as the average change in the probability of the outcome with a one-unit change in the predictor across all values for other variables in the model. While OR are generally preferable when the rate of disease in a sample does not represent the rate of disease in the population, as is the case here for the diagnosed Lyme outcome, we also estimated AME to avoid some known interpretation problems with OR, such as amplifying the magnitude of observed associations [23].

## 3. Results

In total, 324 eligible participants completed the survey, including 185 Lyme patients and 139 non-patients. A total of 65% of participants were female and 32% were male (2 participants declined to identify their sex). The majority of participants (95%) identified as white, 1.9% identified as Latino, and 3.4% identified as other ethnicities (Table 1). In crude analysis, there were no associations between LD and sex, age, or ethnicity; LD diagnosis was associated with pet ownership (OR: 1.81, *p* = 0.013), dog ownership (OR: 1.7, *p* = 0.031), cat ownership (OR: 2.07, *p* = 0.004), and rural residence (OR: 4.925, *p* = 0.001).

In multivariate analyses (controlling for age and sex; Table 2), participants residing in rural areas were more likely to have had an LD diagnosis than their urban/city counterparts (OR = 4.925, *p* = 0.001), with a 33.6% increase in the probability of LD. Pet owners were more likely to have had an LD diagnosis than those who did not own pets (OR: 1.697, *p* = 0.043), with an 11.1% increase in probability (*p* = 0.038). When the type of pet was considered, cat ownership was associated with Lyme diagnosis (OR: 2.143, *p* = 0.007), with cat owners having a 15.7% increase in the probability of having an LD diagnosis (*p* = 0.005), while an association between dog ownership and an LD diagnosis was unsupported (OR: 1.398, *p* = 0.221) (Table 2). Rural residence and cat ownership were independently significantly associated with reported LD diagnosis, but the crude association between Lyme and dog ownership was confounded by rural living.

The majority of participants (270) knew someone with LD, with 80 knowing someone of immediate relation, and 190 knowing someone of non-immediate relation. Participants who reported knowing someone with LD (regardless of relation) were more likely to report wearing insect repellant often (vs. rarely; OR: 6.141, *p* = 0.005), with a 17.0% increase in the probability of wearing insect repellant often (*p* = 0.000) (Table 3). Those knowing someone with LD (regardless of relation) were also more likely to report performing tick checks often (vs. rarely; OR: 7.286, *p* = 0.000), with a 39.2% increase in the probability in performing tick checks often (*p* = 0.000) (Table 3).

There was a trend toward more frequent use of tick prevention across relationships with LD patients, from no relationship to immediate family (Table 3): Participants with an immediate familial connection to someone with LD were more likely to wear insect repellant sometimes (OR: 2.241; *p*: 0.080) or often (OR: 9.300; *p*: 0.001) (vs. rarely); the effect of a non-immediate familial connection was in the same direction but consistently of smaller magnitude than the effect of an immediate familial connection. The pattern was similar for tick checks.

## 4. Discussion

Prior studies have indicated that dogs may serve as a sentinel species for human risk of Lyme disease, as domesticated dogs and humans cohabit similar environments [17,24,25,26,27,28]. Dogs may also increase pet owners’ risk for LD, by bringing ticks inside homes and increasing the amount of time their owners spend outside [24,25,26,27,28].

Very little research, however, has been conducted on the potential for household cats to increase risk for LD in humans. The rural setting of many cat owners may indicate that many of our participants kept at least partially-outdoor cats. Cats allowed outdoors may carry ticks on their bodies into owners’ residences, increasing their risk for tick bites and LD. Cats may roam or travel further than dogs if they are less frequently confined to a fenced yard; so, they may acquire more ticks during their time outdoors than do dogs. Cats also tend to hunt small rodents, indoors and out, and may acquire infected ticks from these prey. We speculate owners may be less likely to use tick prevention (e.g., medicated collars) for cats than dogs and may examine cats for ticks less frequently than dogs, due in part to differences in temperament. Thus, cats, particularly those that are allowed outdoors but also spend time indoors, may increase their owners’ risk for Lyme disease.

Although we did not observe an association between dog ownership and LD, we cannot conclude that no excess risk due to dog ownership exists. Public health messaging advocates for regularly checking dogs for ticks to decrease LD risk for both the dog and the owner. Effective tick-checking among dog owners may have mitigated excess risk for LD associated with dog ownership in this sample, whereas checking cats for ticks may be more difficult.

LD was more common among participants living in rural areas than their urban/city counterparts. This suggests that rural living is a risk factor for the transmission of LD. This is contrary to some recent studies, showing high risk in more suburban and periurban environments [10,12,14].

Controlling for age and sex, rural areas and cat ownership were the two independent predictors of LD identified in our models. Living in rural areas may increase risk for LD as individuals may spend more time outdoors. Pet ownership may also increase LD risk by increasing time spent outdoors; alternatively, pets may increase LD risk by transporting ticks indoors.

As expected, people who knew someone diagnosed with LD reported wearing insect repellant and checking for ticks more frequently than those who did not know someone with LD, and this effect was stronger when the relationship with the LD patient was closer (i.e., immediate family). Prior studies have suggested that raising awareness of the dangers of LD can decrease the number of human cases [29]. Our results are consistent with the idea that when people are aware of the dangers of LD, they act to prevent it. This suggests ample room for improvement with effective public health messaging targeting those with currently low awareness of LD and its prevention.

Limitations of this study include our reliance on self-reports of LD diagnoses. It was not feasible to verify reported diagnoses with medical records; hence, participants may have misunderstood or misrepresented physician diagnoses. Further, this sample may be biased toward those with access to the internet, as the majority of the recruitment for participants was conducted via email and social media. This strategy targeted LD support organizations, so patients may be biased toward those experiencing more severe and prolonged symptoms, and non-patients may be biased toward those who are most aware of LD.

## 5. Conclusions

Understanding ecological, biological, and behavioral risk factors for Lyme disease is critical in helping health agencies and health professionals design and implement the most effective mitigation and prevention strategies. Rural residence and owning a cat are novel risk factors identified within this study, suggesting the need for more research into LD risk factors in the Northeastern United States, as well as public health education about these novel risk factors.

## Figures and Tables

**Table 1:** Sample characteristics (N = 324).

Variable	Lyme Cases (%)	Non-Lyme Cases (%)
**Sex**		
**MALE**	61 (33.0)	41 (29.5)
**FEMALE**	122 (65.9)	95 (69.1)
**OTHER**	2 (1.1)	2 (1.4)
**Age (MEDIAN AND RANGE)**		
**MALE**	54.1 (20–83)	39.8 (18–76)
**FEMALE**	48.3 (18–84)	48.7 (18–81)
**Ethnicity**		
**WHITE**	181 (97.8)	126 (90.5)
L**ATINO/a**	2 (1.1)	4 (2.9)
o**THER**	2 (1.1)	9 (6.6)
**Pet Ownership**		
**CAT**	87 (47.2)	42 (30.2)
**DOG**	118 (63.8)	71 (50.9)
**Community Type**		
R**URAL**	81 (43.8)	17 (12.2)
S**MALL TOWN/VILLAGE**	48 (25.9)	43 (30.9)
S**UBURBAN/LARGE TOWN**	41 (22.2)	60 (43.2)
U**RBAN/CITY**	13 (7.0)	15 (10.8)
O**THER**	2 (1.1)	4 (2.9)
**Insect Repellant Freq.**		
Rarely	92 (49.5)	85 (60.9)
Sometimes	48 (26.1)	35 (25.4)
Often	45 (24.5)	19 (13.8)
**Tick Check Freq.**		
R**ARELY**	26 (14.1)	54 (38.7)
So**METIMES**	27 (14.7)	29 (21.2)
O**FTEN**	132 (71.2)	56 (40.1)

**Table 2:** Odds of a diagnosis with Lyme disease by demographic risk factors, controlling for age and sex.

Variable (Reference)	OR	95% CI	*p*-Value	Average Marginal Effect (AME)	95%CI	*p*-Value
**TYPE OF COMMUNITY** (**U**rban/city)						
Rural/Country	4.925	1.954, 12.413	0.001	0.336	0.132, 0.540	0.001
**S**mall town/village	1.170	0.489, 2.798	0.725	0.039	−0.177, 0.255	0.725
**S**uburb/large town	0.792	0.339, 1.851	0.590	−0.057	−0267, 0.152	0.591
Do**G** Ownership (n**O**)	1.398	0.818, 2.389	0.221	0.071	−0.041, 0.182	0.216
Cat Ownership (n**O**)	2.143	1.232, 3.729	0.007	0.157	0.049, 0.266	0.005

**Table 3:** Odds of using preventive measures of Lyme disease by knowing someone with Lyme disease and degree of relation, controlling for age and sex.

Outcome Variable (Reference)	Predictor Variable (Reference)	OR	95% CI		*p*-Value	Average Marginal Effect (AME)	95% CI	*p*-Value
**Wear Insect** **Repellant (Rarely)**		Know Someone with LD (No)				−0.253	−0.386, −0.120	0.000
Sometimes			2.104	1.006, 4.679	0.051	0.084	−0.038, 0.205	0.176
Often			6.141	1.795, 21.031	0.005	0.170	0.090, 0.250	0.000
**Perform Tick Checks (Rarely)**						−0.336	−0.484, −1.888	0.000
Sometimes			2.179	0.947, 5.013	0.067	−0.056	−0.183, 0.071	0.386
Often			7.286	0.109, 0.803	0.000	0.392	0.257, 0.528	0.000
**Wear Insect Repellant (Rarely)**		Familial Relation						
Sometimes		Non-immediate	2.125	0.965, 4.683	0.061	0.090	−0.036, 0.217	0.160
		Immediate	2.241	0.909, 5.529	0.080	0.062	−0.083, 0.206	0.403
Often		Non-immediate	5.083	1.452, 17.779	0.011	0.139	0.055, 0.223	0.001
		Immediate	9.300	2.507, 34.501	0.001	0.246	0.125, 0.367	0.000
**Perform Tick Checks (Rarely)**		Familial Relation						
Sometimes		Non-immediate	1.954	0.824, 4.632	0.128	−0.047	−0.179, 0.084	0.488
		Immediate	3.258	1.029, 10.309	0.045	0.353	0.212, 0.495	0.000
Often		Non-immediate	5.847	2.710, 12.604	0.000	−0.075	−0.218, 0.067	0.300
		Immediate	14.041	5.145, 34.501	0.000	0.486	0.329, 0.643	0.000

## Data Availability

The data presented in this study are available on request from the corresponding author. The data are not publicly available due to privacy concerns.

## Supplementary Materials

The following supporting information can be downloaded at: https://www.mdpi.com/article/10.3390/ijerph19095618/s1, Survey.

## References

[1] Burgdorfer W., Barbour A.G., Hayes S.F., Benach J.L., Grunwaldt E., Davis J.P. (1982). Lyme Disease—A Tick-Borne Spirochetosis?. Science.

[2] Eisen R.J., Piesman J., Zielinski-Gutierrez E., Eisen L. (2012). What Do We Need to Know About Disease Ecology to Prevent Lyme Disease in the Northeastern United States?. J. Med. EÈntomol..

[3] Shadick N.A., Phillips C.B., Sangha O., Logigian E.L., Kaplan R.F., Wright E.A., Fossel A.H., Fossel K., Berardi V., Lew R.A. (1999). Musculoskeletal and Neurologic Outcomes in Patients with Previously Treated Lyme Disease. Ann. Intern. Med..

[4] Shadick N.A., Phillips C.B., Logigian E.L., Steere A.C., Kaplan R.F., Berardi V.P., Duray P.H., Larson M., Wright E.A., Ginsburg K.S. (1994). The Long-Term Clinical Outcomes of Lyme Disease: A Population-Based Retrospective Cohort Study. Ann. Intern. Med..

[5] Citera M., Freeman P.R., Horowitz R.I. (2017). Empirical validation of the Horowitz Multiple Systemic Infectious Disease Syndrome Questionnaire for suspected Lyme disease. Int. J. Gen. Med..

[6] Rebman A.W., Bechtold K.T., Yang T., Mihm E.A., Soloski M.J., Novak C.B., Aucott J.N. (2017). The Clinical, Symptom, and Quality-of-Life Characterization of a Well-Defined Group of Patients with Posttreatment Lyme Disease Syndrome. Front. Med..

[7] Kostic T., Momčilović S., Perišić Z., Apostolovic S., Cvetković J., Jovanović A., Barac A., Salinger S., Otašević S. (2017). Manifestations of Lyme carditis. Int. J. Cardiol..

[8] Schwartz A.M., Kugeler K.J., Nelson C.A., Marx G.E., Hinckley A.F. (2021). Evaluation of commercial insurance claims as an annual data source for Lyme disease diagnoses. Emerg. Infect. Dis..

[9] Orioski K.A., Campbell G.L., Genese C.A., Beckley J.W., Schriefer M.E., Spitalny K.C., Dennis D.T. (1998). Emergence of Lyme Disease in Hunterdon County, New Jersey, 1993: A Case-Control Study of Risk Factors and Evaluation of Reporting Patterns. Am. J. Epidemiol..

[10] Allan B.F., Keesing F., Ostfeld R.S. (2003). Effect of Forest Fragmentation on Lyme Disease Risk. Conserv. Biol..

[11] Brownstein J.S., Skelly D.K., Holford T.R., Fish D. (2005). Forest fragmentation predicts local scale heterogeneity of Lyme disease risk. Oecologia.

[12] Roome A., Spathis R., Hill L., Darcy J.M., Garruto R.M. (2018). Lyme Disease Transmission Risk: Seasonal Variation in the Built Environment. Healthcare.

[13] Barbour A.G., Fish D. (1993). The Biological and Social Phenomenon of Lyme Disease. Science.

[14] Diuk-Wasser M.A., Brinkerhoff R., Kitron U., Fish D., Melton F., Cislo P., Tsao J.I., Rowland M., Barbour A., Vourc’H G. (2012). Human Risk of Infection with Borrelia burgdorferi, the Lyme Disease Agent, in Eastern United States. Am. J. Trop. Med. Hyg..

[15] LoGiudice K., Ostfeld R.S., Schmidt K.A., Keesing F. (2003). The ecology of infectious disease: Effects of host diversity and community composition on Lyme disease risk. Proc. Natl. Acad. Sci. USA.

[16] Lane R.S., Steinlein D.B., Mun J. (2004). Human Behaviors Elevating Exposure to *Ixodes pacificus* (Acari: Ixodidae) Nymphs and Their Associated Bacterial Zoonotic Agents in a Hardwood Forest. J. Med. EÈntomol..

[17] Finch C., Al-Damluji M.S., Krause P.J., Niccolai L., Steeves T., O’Keefe C.F., Diuk-Wasser M.A. (2014). Integrated Assessment of Behavioral and Environmental Risk Factors for Lyme Disease Infection on Block Island, Rhode Island. PLoS ONE.

[18] Goossens H.A.T., van den Bogaard A.E., Nohlmans M.K.E. (2001). Dogs as Sentinels for Human Lyme Borreliosis in The Netherlands. J. Clin. Microbiol..

[19] Carroll J.F., Kramer M. (2001). Different Activities and Footwear Influence Exposure to Host-Seeking Nymphs of Ixodes scapularisandAmblyomma americanum(Acari: Ixodidae). J. Med. EÈntomol..

[20] Brewer N.T., Weinstein N.D., Cuite C.L., Herrington J.E. (2004). Risk perceptions and their relation to risk behavior. Ann. Behav. Med..

[21] Shadick N.A., Daltroy L.H., Phillips C.B., Liang U.S., Liang M.H. (1997). Determinants of tick-avoidance behaviors in an endemic area for Lyme dis-ease. Am. J. Prev. Med..

[22] Ley C., Olshen E.M., Reingold A.L. (1995). Case-Control Study of Risk Factors for Incident Lyme Disease in California. Am. J. Epidemiol..

[23] Norton E.C., Dowd B.E. (2018). Log Odds and the Interpretation of Logit Models. Health Serv. Res..

[24] Bowser N.H., Anderson N.E. (2018). Dogs (Canis familiaris) as Sentinels for Human Infectious Disease and Application to Canadian Populations: A Systematic Review. Vet. Sci..

[25] Hamer S.A., Tsao J.I., Walker E.D., Mansfield L.S., Foster E.S., Hickling G.J. (2009). Use of tick surveys and serosurveys to evaluate pet dogs as a sentinel species for emerging Lyme disease. Am. J. Vet. Res..

[26] Lindenmayer J.M., Marshall D., Onderdonk A.B. (1991). Dogs as sentinels for Lyme disease in Massachusetts. Am. J. Public Health.

[27] Little S.E., Heise S.R., Blagburn B.L., Callister S.M., Mead P.S. (2010). Lyme borreliosis in dogs and humans in the USA. Trends Parasitol..

[28] Smith F.D., Ballantyne R., Morgan E., Wall R. (2012). Estimating Lyme disease risk using pet dogs as sentinels. Comp. Immunol. Microbiol. Infect. Dis..

[29] Sharareh N., Sabounchi N.S., Roome A., Spathis R., Garruto R.M. (2017). Model-based risk assessment and public health analysis to prevent Lyme disease. R. Soc. Open Sci..

