# Supplementary material for: Cat Ownership and Rural Residence Are Associated with Lyme Disease Prevalence in the Northeastern United States
Source: Int J Environ Res Public Health. 2022 May 5;19(9):5618. doi: 10.3390/ijerph19095618 (PMC9105077; doi:10.3390/ijerph19095618)
Supplement: Supplementary file 1 [file ijerph-19-05618-s001.zip › ijerph-1659000-supplementary.pdf]

Participant (Subject) #: _____________

UNDERSTANDING THE LIFESTYLE IMPACTS OF LYME DISEASE SURVEY FORM

Title of research project: Understanding the Impact of Emerging Infectious Diseases in Human Populations: the case for Lyme disease

---

SECTION A

1. What gender are you?
   ◯ Male ◯ Female

2. What is your age? _____________

3. What state do you live in? _______________

4. What county do you live in? ___________________

5. What kind of community do you live in?
   ◯ Urban/city ◯ Sub-urban/large town ◯ Small town/village
   ◯ Rural/country ◯ Other (please specify): ____________

6. What is your primary occupational environment?
   ◯ Farm/agriculture ◯ Office Building ◯ Work from home
   ◯ Factory work ◯ School ◯ Forestry/park service
   ◯ Hospital/healthcare ◯ Machine shop/mechanic ◯ Construction
   ◯ Animal/Veterinary clinic ◯ Quarry/gas drilling
   ◯ Other (please specify): ____________

7. How do you describe your ethnicity?
   ◯ White (Caucasian) ◯ African American ◯ Australasian
   ◯ Asian ◯ African ◯ Hawaiian/Pacific Islander
   ◯ Latino ◯ Other (please specify): ____________

8. Do you have any household pets? (i.e., pets that live INSIDE your home?)
   ◯ Yes ◯ No

9. If you own a household pet, what pets do you own? Check all that apply
   ◯ Not applicable ◯ Dog(s) ◯ Cat(s)
   ◯ Other (please specify): _____________

Participant (Subject) #: _______________

10. How often do you exercise?
   ◯Never ◯Less than once per month ◯1-3 times per month
   ◯1-3times per week ◯4-6 times per week ◯Everyday

11. How often do you engage in social activities (i.e. go out with friends, etc.)?
   ◯Never ◯Less than once per month ◯1-3 times per month
   ◯1-3 times per week ◯4-6 times per week ◯Everyday

12. How many meals do you typically eat per day?
   ◯1 meal ◯2 meals ◯3 meals
   ◯4 meals ◯5 meals ◯6+ meals

13. How many hours do you typically sleep in a 24 hour period?
   ◯1-3 ◯4-6 ◯7-9
   ◯10-12 ◯13-15 ◯16+

14. How often do you feel tired or lethargic after a typical night sleep?
   ◯Never ◯Rarely ◯Sometimes
   ◯Often ◯Always

15. How would you categorize your overall health?
   ◯Very Poor ◯Poor ◯Neither good nor poor
   ◯Good ◯Very good

16. How would you assess your overall health compared to that of others of your own age?
   ◯Much worse ◯Slightly worse ◯Neither better nor worse
   ◯Slightly better ◯Much better

17. How often do you feel fearful?

	1	2	3	4	5	
Always	◯	◯	◯	◯	◯	Never

18. How often do you feel angry?

	1	2	3	4	5	
Always	◯	◯	◯	◯	◯	Never

19. How often do you feel sad?

	1	2	3	4	5	
Always	◯	◯	◯	◯	◯	Never

20. How often do you feel happy?

	1	2	3	4	5	
Always	◯	◯	◯	◯	◯	Never

21. How often do you feel anticipation?

	1	2	3	4	5	
Always	◯	◯	◯	◯	◯	Never

22. On average, how many times per year do you visit the doctor (primary care physician)?
    ◯0 ◯1-2 ◯3-5 ◯6-8
    ◯9-11 ◯12-14 ◯15+

23. Do you know someone (other than yourself) that has been diagnosed with Lyme disease (by a physician)?
    ◯Yes ◯No

24. If you know someone that's been diagnosed with Lyme disease, what is their relation to you? Check all that apply.
    ◯Not applicable ◯Spouse ◯Child
    ◯Sibling ◯Parent ◯Relative (not immediate)
    ◯Friend ◯Acquaintance
    ◯Other (please specify): ____________

25. How often do you wear bug spray (containing DEET) when you are outside (i.e. gardening, hiking, walking, etc.)?
    ◯Never ◯Rarely ◯Sometimes
    ◯Often ◯Always

26. How often do you check yourself for ticks after being outside (i.e. gardening, hiking, walking, etc.)?
    ◯Never ◯Rarely ◯Sometimes
    ◯Often ◯Always

27. Have you ever been diagnosed with Lyme disease by a physician?
    ◯Yes ◯No

(If NO, please continue to SECTION C. If YES, please continue to SECTION B)

SECTION B

28. When were you diagnosed with Lyme disease (for the first time if you've had multiple diagnoses) (year; i.e. 2003)? __________________

29. If you have been diagnosed with Lyme, were you treated by a physician?
    ◯Yes ◯No

30. Did your symptoms clear after initial treatment?
    ◯Yes ◯No

31. If your symptoms did NOT clear after initial treatment, were you treated again?
    ◯Not applicable ◯Yes ◯No

32. Approximately how long did your symptoms last from the time you were first diagnosed with Lyme disease?
    ◯1-2weeks ◯3-4 weeks ◯2-4 months ◯4-6 months
    ◯6-8 months ◯8-10 months ◯10-12 months ◯1-2 years
    ◯2-5 years ◯5+ years ◯My symptoms are still occurring

33. What symptoms did you experience? Check all that apply.
    ◯Erythema migrans rash ◯Fever ◯Knee joint pain
    ◯Hip joint pain ◯Ankle joint pain ◯Elbow joint pain
    ◯Shoulder joint pain ◯Wrist joint pain ◯Other joint pain
    ◯Neck pain ◯Muscle weakness ◯Leg muscle cramps
    ◯Back muscle cramps ◯Arm muscle cramps ◯Other muscle cramps
    ◯Facial tingling ◯Facial paralysis ◯Impaired memory
    ◯Sore throat ◯Unusual hair loss ◯Difficulty comprehending
    ◯Absent mindedness ◯Confusion ◯Dizziness
    ◯Light headedness ◯Misspeaking ◯Anxiousness
    ◯Unusual fatigue ◯Mood swings ◯Nausea

◯Diarrhea ◯Heart burn

◯Other (please specify): ____________

34. Were you ever diagnosed with Post-Treatment Lyme Disease Syndrome by a physician?
 ◯Yes ◯No

35. Can you recall ever being bitten by a tick?
 ◯Yes ◯No

36. How often did you exercise **BEFORE** being diagnosed with Lyme disease?
 ◯Never ◯Less than once per month ◯1-3 times per month

 ◯1-4times per week ◯4-6 times per week ◯Everyday

37. How often do you exercise now, **AFTER** being diagnosed with Lyme disease?
 ◯Never ◯Less than once per month ◯1-3 times per month

 ◯1-3 times per week ◯4-6 times per week ◯Everyday

38. How often did you engage in social activities (i.e. go out with friends, etc.) **BEFORE** being diagnosed with Lyme disease?
 ◯Never ◯Less than once per month ◯1-3 times per month

 ◯1-3 times per week ◯4-6 times per week ◯Everyday

39. How often do you engage in social activities (i.e. go out with friends, etc.) now, **AFTER** being diagnosed with Lyme disease?
 ◯Never ◯Less than once per month ◯1-3 times per month

 ◯1-3 times per week ◯4-6 times per week ◯Everyday

40. How many meals did you typically eat per day **BEFORE** being diagnosed with Lyme disease?
 ◯1 meal ◯2 meals ◯3 meals

 ◯4 meals ◯5 meals ◯6+ meals

41. How many meals do you typically eat per day now, **AFTER** being diagnosed with Lyme disease?
 ◯1 meal ◯2 meals ◯3 meals

 ◯4 meals ◯5 meals ◯6+ meals

42. How many hours did you typically sleep in a 24 hour period **BEFORE** being diagnosed with Lyme disease?

◯1-3 ◯4-6 ◯7-9

◯10-12 ◯13-15 ◯16+

43. How many hours do you typically sleep in a 24 hour period now, **AFTER** being diagnosed with Lyme disease?

◯1-3 ◯4-6 ◯7-9

◯10-12 ◯13-15 ◯16+

44. How often did you feel tired or lethargic after a typical night sleep **BEFORE** being diagnosed with Lyme disease?

◯Never ◯Rarely ◯Sometimes

◯Often ◯Always

45. How often do you feel tired or lethargic after a typical night sleep now, **AFTER** being diagnosed with Lyme disease?

◯Never ◯Rarely ◯Sometimes

◯Often ◯Always

46. How would you categorize your overall health **BEFORE** being diagnosed with Lyme disease?

◯Very Poor ◯Poor ◯Neither good nor poor

◯Good ◯Very good

47. How would you categorize your overall health **AFTER** being diagnosed with Lyme disease?

◯Very Poor ◯Poor ◯Neither good nor poor

◯Good ◯Very good

48. How would you assess your overall health compared to that of others of your own age **BEFORE** being diagnosed with Lyme disease?

◯Much worse ◯Slightly worse ◯Neither better nor worse

◯Slightly better ◯Much better

49. How would you assess your overall health compared to that of others of your own age **AFTER** being diagnosed with Lyme disease?

◯Much worse ◯Slightly worse ◯Neither better nor worse

◯Slightly better ◯Much better

50. How often did you feel fearful **BEFORE** being diagnosed with Lyme disease?

	1	2	3	4	5	
Always	◯	◯	◯	◯	◯	Never

51. How often do you feel fearful now, **AFTER** being diagnosed with Lyme disease?

	1	2	3	4	5	
Always	◯	◯	◯	◯	◯	Never

52. How often did you feel angry **BEFORE** being diagnosed with Lyme disease?

	1	2	3	4	5	
Always	◯	◯	◯	◯	◯	Never

53. How often do you feel angry now, **AFTER** being diagnosed with Lyme disease?

	1	2	3	4	5	
Always	◯	◯	◯	◯	◯	Never

54. How often did you feel sad **BEFORE** being diagnosed with Lyme disease?

	1	2	3	4	5	
Always	◯	◯	◯	◯	◯	Never

55. How often do you feel sad now, **AFTER** being diagnosed with Lyme disease?

	1	2	3	4	5	
Always	◯	◯	◯	◯	◯	Never

56. How often did you feel happy **BEFORE** being diagnosed with Lyme disease?

	1	2	3	4	5	
Always	◯	◯	◯	◯	◯	Never

57. How often do you feel happy now, **AFTER** being diagnosed with Lyme disease?

	1	2	3	4	5	
Always	◯	◯	◯	◯	◯	Never

58. How often did you feel anticipation **BEFORE** being diagnosed with Lyme disease?

	1	2	3	4	5	
Always	◯	◯	◯	◯	◯	Never

59. How often do you feel anticipation now, **AFTER** being diagnosed with Lyme disease?

	1	2	3	4	5	

Always ◯ ◯ ◯ ◯ ◯ Never

60. On average, how many times per year did you visit the doctor (primary care physician or specialist) **BEFORE** being diagnosed with Lyme disease?
◯0 ◯1-2 ◯3-5 ◯6-8

◯9-11 ◯12-14 ◯15+

61. On average, how many times per year do you visit the doctor (primary care physician or specialist) now, **AFTER** being diagnosed with Lyme disease?
◯0 ◯1-2 ◯3-5 ◯6-8

◯9-11 ◯12-14 ◯15+

62. How often did you wear bug spray (containing DEET) when you are outside (i.e. gardening, hiking, walking, etc.) **BEFORE** being diagnosed with Lyme disease?
◯Never ◯Rarely ◯Sometimes

◯Often ◯Always

63. How often do you wear bug spray (containing DEET) when you are outside (i.e. gardening, hiking, walking, etc.) **AFTER** being diagnosed with Lyme disease?
◯Never ◯Rarely ◯Sometimes

◯Often ◯Always

64. How often did you check yourself for ticks after being outside (i.e. gardening, hiking, walking, etc.) **BEFORE** being diagnosed with Lyme disease?
◯Never ◯Rarely ◯Sometimes

◯Often ◯Always

65. How often do you check yourself for ticks after being outside (i.e. gardening, hiking, walking, etc.) **AFTER** being diagnosed with Lyme disease?
◯Never ◯Rarely ◯Sometimes

◯Often ◯Always

SECTION C

66. Would you like to receive an email update when data analyses are complete?
    a. Yes (please provide email address): ______________________________
    b. No

Thank you for participating in this survey. The information you provide will assist in gaining a better understanding of Lyme disease and the impact it has on human health.

Should you have any questions, please do not hesitate to contact Amanda Roome at aroome1@binghamton.edu.
